# Predator-Induced Morphological Plasticity Across Local Populations of a Freshwater Snail

**DOI:** 10.1371/journal.pone.0021773

**Published:** 2011-07-19

**Authors:** Christer Brönmark, Thomas Lakowitz, Johan Hollander

**Affiliations:** Aquatic Ecology Unit, Department of Biology, University of Lund, Lund, Sweden; University of Western Ontario, Canada

## Abstract

The expression of anti-predator adaptations may vary on a spatial scale, favouring traits that are advantageous in a given predation regime. Besides, evolution of different developmental strategies depends to a large extent on the grain of the environment and may result in locally canalized adaptations or, alternatively, the evolution of phenotypic plasticity as different predation regimes may vary across habitats. We investigated the potential for predator-driven variability in shell morphology in a freshwater snail, *Radix balthica*, and whether found differences were a specialized ecotype adaptation or a result of phenotypic plasticity. Shell shape was quantified in snails from geographically separated pond populations with and without molluscivorous fish. Subsequently, in a common garden experiment we investigated reaction norms of snails from populations' with/without fish when exposed to chemical cues from tench (*Tinca tinca*), a molluscivorous fish. We found that snails from fish-free ponds had a narrow shell with a well developed spire, whereas snails that coexisted with fish had more rotund shells with a low spire, a shell morphology known to increase survival rate from shell-crushing predators. The common garden experiment mirrored the results from the field survey and showed that snails had similar reaction norms in response to chemical predator cues, i.e. the expression of shell shape was independent of population origin. Finally, we found significant differences for the trait means among populations, within each pond category (fish/fish free), suggesting a genetic component in the determination of shell morphology that has evolved independently across ponds.

## Introduction

Phenotypic plasticity is an important strategy among prey organisms against predation in freshwater habitats and there are many examples of plasticity in behavioural, chemical and morphological defence traits [1]–[5]. The evolution of phenotypically plastic defence traits is favoured when prey have reliable cues of detecting the presence of a predator, when the inducible defence provides a benefit in terms of increased survival probability in the presence of predators but incurs a fitness cost in their absence and, further, when there is a high spatial or temporal variability in predation pressure [6].

Environmental heterogeneity could be either fine-grained or course-grained [7], where an organism living in a fine-grained environment encounters numerous habitats, and in a coarse-grained environment only a single habitat, during its lifetime. The optimal developmental strategy among prey organisms – e.g. constitutive traits versus phenotypic plasticity – depends accordingly on the environmental grain of predators. Hence, a coarse grain environment is expected to select for canalisation and result in locally adapted ecotypes, while a fine grain environment with large environmental heterogeneity would favour the evolution of phenotypic plasticity or a single generalist [7], [8]. However, even though canalisation is expected in coarse-grain environments, gene flow among populations may counteract gene frequency changes due to divergent selection and thus impose a limit on local adaption [9], [10]. Thus, organisms that possess high dispersal rates would generally experience a fine-grained environment and evolve phenotypic plasticity to ensure adaptation to a fluctuating or variable environment [8], [11], [12]. In freshwater habitats, the expression of plastic anti-predator traits may vary on a spatial scale between discrete habitat units such as ponds or lakes due to differences in the predator assemblage. Within a pond, temporal variability in predation pressure, e.g. extinction/colonisation cycles of predatory fish, would create a fine-grained environment favouring the development of phenotypic plasticity in invertebrate prey organisms. However, permanent ponds typically demonstrate consistency in presence/absence of fish over many generations of the prey organism (Brönmark, personal observation) and are thus a homogenous, coarse-grained environment from the point of view of predation regime. However, a high dispersal rate among prey populations – between ponds with different predation regimes – may create a fine-grained environment and here selection should favour the evolution of phenotypic plasticity [8].

Here, we explore the potential for predator-driven differences in shell shape in a fresh water snail, *Radix balthica*, (formerly *Lymnaea peregra*; [13]). Snails occur in all types of freshwater habitats from small ephemeral ponds and streams to large lakes and rivers and are exposed to predation from a range of different predators [14]–[16]. Freshwater snails are model organisms for studying traits that have evolved as a measure against predation and a number of studies have shown that freshwater snails have evolved a diverse set of anti-predator adaptations, including behaviour [5], [17], [18] and morphology [19]–[24].

*Radix balthica* is known to show considerable morphological variation in shell shape [25], from elongated shells with narrow apertures to more round shells with wider apertures. Earlier studies have related shell shape to differences in abiotic factors among habitats [25]–[27]. In this study, we first investigate if geographical differences in shell shape of *R. balthica* in pond populations were related to the prevailing regime in a pond, i.e. presence or absence of molluscivorous fish. We hypothesized that *R. balthica* from ponds with molluscivorous fish would have a more rotund shell shape as this is known to reduce predation efficiency from shell-crushing predators (19). We then performed a common garden experiment to investigate if found differences were due to constitutive traits or phenotypic plasticity and if there were genetic differences in trait means and variation in the magnitude of plasticity.

## Materials and Methods

### Field survey

To investigate the relationship between snail shell shape and presence of fish predators we conducted a field survey of 22 permanent ponds of similar size and morphology across the province of Skåne, southern Sweden, chosen from a large pool of ponds surveyed for fauna and flora [28]–[30]. Eleven of the ponds contained molluscivorous fish, either tench (*Tinca tinca*) or crucian carp (*Carassius carassius*), whereas the other eleven ponds had no fish. *R. balthica* was collected in the ponds by sweep-netting in the littoral zone and preserved in alcohol. The number of snails sampled from each pond type ranged from 5–12 (fish free ponds; mean 9.4±2.2, 1 SD, fish ponds; mean 8.1±2.6, 1 SD). For morphometric measurements, the snail shell was placed with the opening facing down on a flatbed scanner (Epson 2450 Photo) and the images were then analyzed using an image analyzing program (SHAPE; [31]). In order to assess shape variation among populations from fish and fish free ponds we fit a model with “Pondtype” and “Population” nested under Pondtype in an analysis of variance with the dependent variables PC1 and PC2, describing shape variation.

### Common garden experiment

To understand the variation of shape traits among *R. balthica* populations exposed to different predation regimes (fish/no fish), we did a common garden experiment where F1 snails from a subset of the populations from the field survey were used. Snails from 5 fish free and 4 fish ponds were grown in presence or absence of predator cues. From each population, 10–20 snails were collected and brought back to the laboratory. Snails were placed in 10 litre plastic aquaria, one population per aquarium, allowed to reproduce and three weeks after the snail eggs had hatched we collected the juvenile snails to be used in the experiments. Tench were collected by electrofishing in Lake Krankesjön, 20 km east of Lund, southern Sweden. Tench biomass was 116.2 g±37.5 g (1 SD). The tench in the fish treatment tanks were fed 6 adult *R. balthica* per week.

The experimental setup consisted of 18 large (70 litre) opaque plastic tanks containing either no fish (control) or two tench (fish cue). Each tank was stocked with 20 snails from a specific population. The snails were divided up into two 2 litre containers (10 snails per container) that were submerged in the larger tank. The small containers had two holes (10 cm in diameter) fitted with net (mesh size: 0.5 mm) to allow water exchange. The experiment was kept at a light∶dark cycle of 12∶12 h and water temperature varied from 19–21°C. After 12 weeks we terminated the experiment and snails were deep-frozen. At a later date the snails were thawed, soft tissues removed and shells were scanned on a flatbed scanner. Snail lengths were measured (Image J; control: 8.7±1.0 mm (mean ±1 SD), fish treatment: 7.8±2.3 mm). The outline shell shape was analysed as described below. We examined the data with an analysis of variance including the factors “Treatment” with two levels: fish cue and control; “Pondtype” with two levels: fish or fish free ponds; “Population” nested under Pondtype; the interaction term between Treatment×Pondtype and finally: Treatment×Population nested under Pondtype, with the dependent variables PC1 and PC2. All statistical analysis was performed in R [32].

The study complies with the current laws in Sweden; ethical concerns on care and use of experimental animals were followed under the permission approved for this study (M165-07) from the Malmö/Lund Ethical Committee.

### *Shape analysis*

Since snails have very few homologous points that can be used in landmark morphometrics, we chose to use elliptic Fourier analysis as it captures the outline of the shell and thus the curved shapes that are responsible for an increase in shell roundness. The analysis is independent of size, position and rotation of the object, variables not associated to shape. The program SHAPE creates a contrast between the object and the background and read the contour of the object by edge detection. Furthermore, the program generates principal components related to shape characteristics, and the scores of principal components can be stored and exported for subsequent analysis in additional software (see [31] for a complete description).

**Figure 1:**
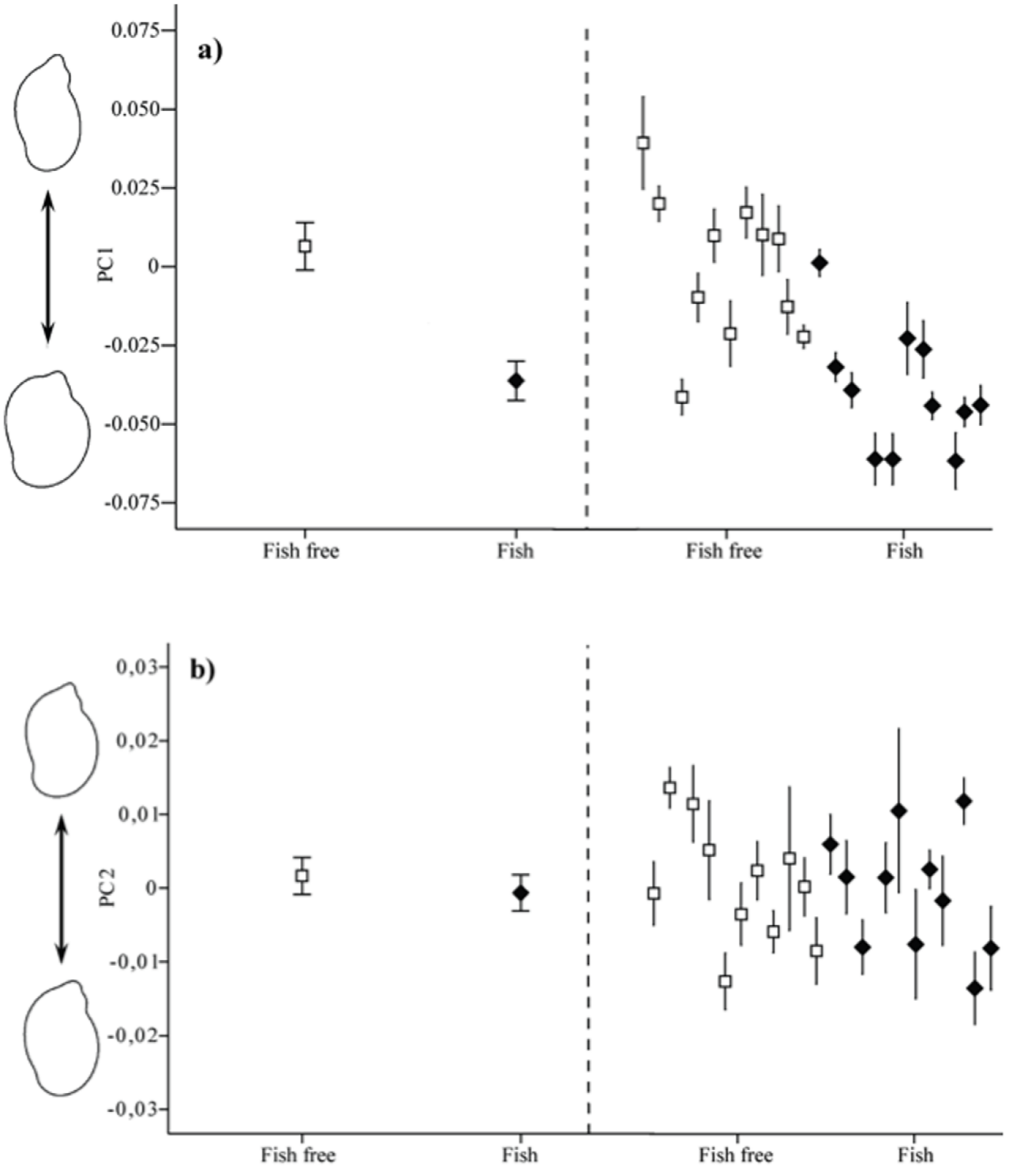
Shell morphology in *Radix balthica* from the field survey. Shell shape was analysed with shell outline analyses and shape characteristics are expressed as principal component scores (PC 1, a; PC 2,b) with the visualized shapes to the left. Mean shell shapes for ponds without (open squares) and with (closed diamonds) molluscivorous fish are shown to the left of the broken, vertical line, whereas shell shapes from each separate population are shown to the right. Error bars indicate one standard error.

**Figure 2:**
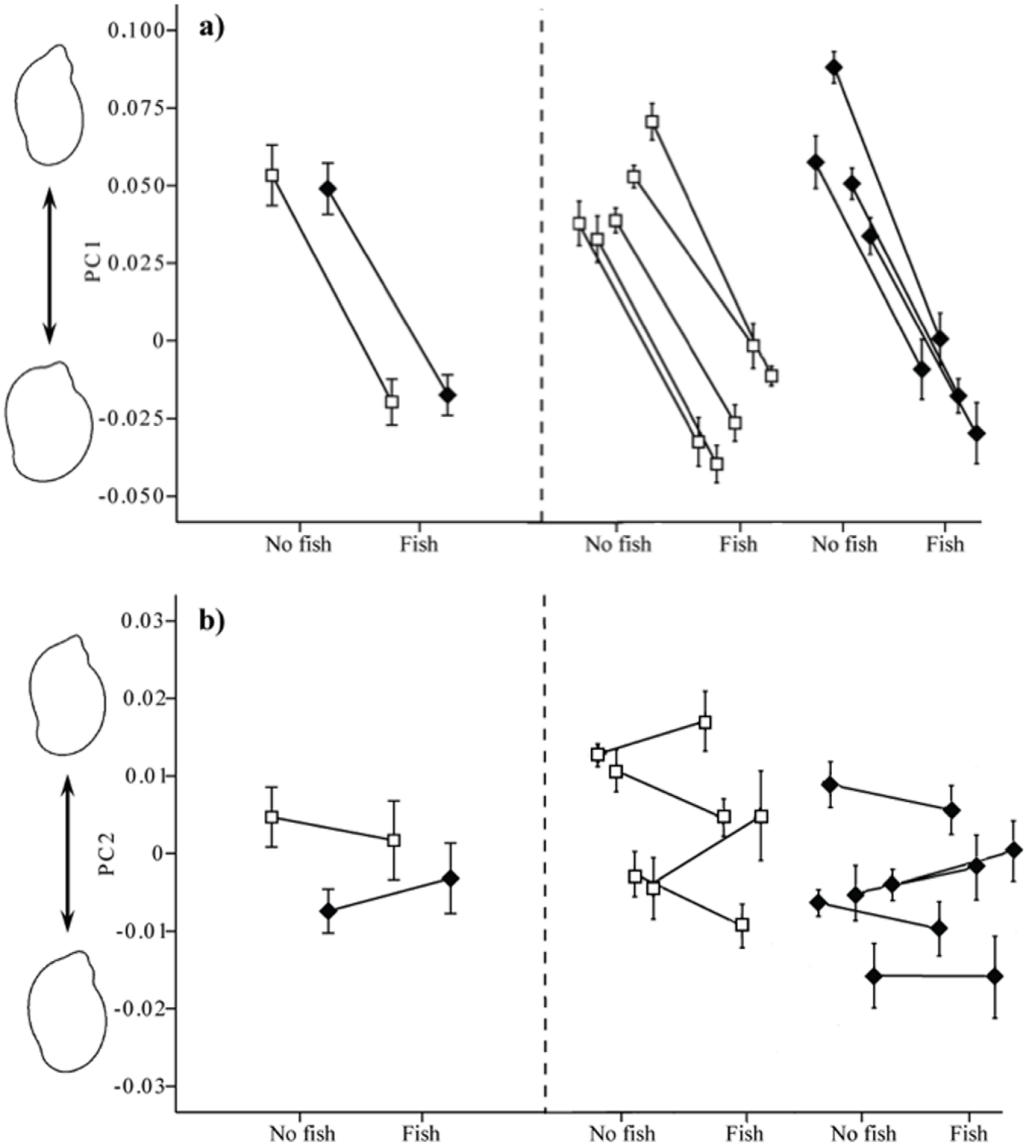
Reaction norms of shell morphology in *Radix balthica* from the common garden experiment. Shell shape was analysed with shell outline analyses and shape characteristics are expressed as principal component scores (PC 1, a; PC 2,b) with the visualized shapes to the left. Mean shell shapes for offspring from ponds without (open squares) or with (closed diamonds) molluscivorous fish, raised either in the presence or absence of chemical cues from tench are shown to the left of the broken, vertical line, whereas the reaction norms for each separate population are shown to the right. Error bars indicate one standard error.

Snails from the field survey and the common garden experiment were analyzed together so that shape (PC scores) of snails from the field could be compared directly with the shape of snails from the experiment. Principal components explaining at least 5% of the variation in shell shape (PC1 and PC2) was analysed as the dependent variable in both the field survey and the common garden experiment. To visualize shape variations we used inverse Fourier transformation to produce an image of the shell. This image shows the outline shape of the shell and has to be interpreted visually for each principal component (see outline snail images in Figs. 1 and 2).

## Results

In the principal components analysis of shell shape, the cumulative contribution of the first two components made up 79.2% of the variation. The first principal component, which accounted for 71.1% of the variation in shell shape, is associated with the wideness of the body whorl and the size of the shell opening, as well as the relative height of the apex. Negative scores are generated when the shell has a wider body whorl, resulting in a rounder shell shape and a relatively lower spire, whereas positive scores are associated with a shell that has a narrow body whorl as well as a narrower opening and a well developed spire (Fig. 1a and 2a). The second principal component, which accounts for 8.1% of the variation in shell shape, is associated with a narrowing of the second to last whorl for negative scores and a widening of this area for positive scores (Fig. 1b and 2b). See Appendix S1 for further details of the principal components analysis.

### Field survey

There was a significant effect of predation regime on the first principal component (*F*_1, 20_ = 112.0, *p*<0.001), i.e. snails from ponds with molluscivorous fish had a rounder shell shape and a lower spire than snails from ponds without fish (Fig. 1a). The second principal component did not differ between pond categories (*F*_1, 20_ = 1.1, *p* = 0.30; Fig. 1b). There was also a significant difference in shape among populations within each pond category (fish/no fish ponds); the effect of populations nested within pond categories was significant for both PC 1 (*F*_20, 170_ = 6.0, *p*<0.001) and PC 2 (*F*_20, 170_ = 2.8, *p*<0.001).

### Common garden experiment

There was no significant effect of pond category (fish/no fish ponds) on shell shape as explained by the first principal component (*F*_1, 7_ = 0.06, *p* = 0.98), whereas there was a highly significant effect of treatment (fish/no fish cues; *F*_1, 7_ = 467.2, *p*<0.001). Snails exposed to fish cues had a rounder body shape than control snails (fish/no fish). There was no significant treatment by pond category interaction (*F*_1, 7_ = 0.71, *p* = 0.40) indicating that snails responded similarly to fish cues independent of the predation regime in the pond they originated from (Fig. 2a). However, there was a significant effect of population nested within pond category (*F*_7, 144_ = 11.7, *p*<0.001) suggesting that there are differences among populations within pond category in how snails react to experimental treatments. For the second principal component there was no effect of treatment (*F*_1, 7_ = 0.09, *p* = 0.76), whereas there was a significant effect of pond category (*F*_1, 7_ = 22.2, *p*<0.001) due to a widening of the second to last whorl in snails from ponds without fish in the absence of fish cues. There was also a significant treatment by pond category interaction (*F*_1, 7_ = 4.8, *p* = 0.03) and a significant effect of population nested within pond category (*F*_7, 144_ = 12.8, *p*<0.001; Fig. 2b).

## Discussion

*Radix balthica* has for long been known for large interpopulation variation in shell morphology, ranging from individuals with a relatively small aperture, high spire and a slow expanding body whorl to individuals with a large aperture, low spire and a rapidly growing body whorl [25]. For that reason, the species was previously divided into two species, *Lymnaea peregra* and *L. ovata* [13], later Wullschleger and Jokela [33] found that the shell form of *L. peregra* and *L. ovata* converged after two generations in the lab and argued that differences in shell shape was a result of phenotypic plasticity in response to habitat differences in permanence and water movement.

Shell shape has been assessed previously in freshwater snails [19], [34], although, few studies have compared differences in shell morphology among several populations over a geographical range. In this survey, we found significant differences in average shell morphology between ponds with and without molluscivorous fish. In ponds without fish, *R. balthica* typically had narrow elongated shells with an accentuated spire, whereas in ponds with molluscivorous fish shells had a more rounded shell form and a low spire, a shape evidently associated with adaptation against fish predation [19], [35].

In recent years, a number of studies have shown that prey organism may respond to the threat of predation by modifying phenotypically plastic traits [2], [6], [36]–[40]. In our common garden experiment we were able to show that a significant part of the variability in the shell morphology is due to a plastic response induced by chemical cues from molluscivorous fish. Interestingly, the change in shell morphology demonstrated in the common garden experiment was in the same direction as found in the field survey and thus argues for a plasticity defence strategy against molluscivorous fish in the wild. Furthermore, the common garden experiment impose reaction norms in the same direction independent of predation regime in the pond of origin, a result similar to what Trussel [37] and Hollander et al. [38] found in *Littorina obtusata* and *L. saxatilis*. For example, Trussel [37] found that different populations in *L. obtusata* showed similar plasticity in shell thickness, suggesting that the reaction norm in the different populations had evolved similar slopes even though the populations had been in contact with the predator for different periods, which may indicate that the evolution of phenotypic plasticity is rapid. Nevertheless, in the grand trait mean for *R. balthica* (fig. 2a, the average phenotype across all populations) there was no difference between snails originating from fish and no-fish ponds, while within each group, a substantial amount of variation in trait means was exposed, demonstrating genetic differences among populations within treatments. Such differences represent genetic variation [43] and may illustrate phenotypic variation around an adaptive optimum, and if the optimum for the most favourable phenotype fluctuates spatially or temporally, the adaptive value will vary across populations [40], [41]. In spite of gene flow among ponds (see below), genetic differences are conserved to some extent and may illustrate strong natural selection to favour certain local adaptation or, alternatively, be a result of genetic drift.

A key issue regarding the model system of *R. balthica* is the reason the species has evolved a developmental strategy as phenotypic plasticity and not constitutive traits, since the ponds we have surveyed show a homogeneous milieu in terms of predation or no-predation. Theoretical models suggest that plasticity is favored in heterogeneous or fluctuating environments, whereas in stable environments there will be a loss of plasticity and genetic assimilation of traits, i.e. due to directional selection traits will become genetically determined and canalized, resulting in flat reaction norms [12], [42]. However, high gene flow among habitats may reduce the process of genetic assimilation and adaptive divergence and Sultan and Spencer [12] predicted that plasticity would be favoured by high gene flow in a landscape with metapopulation structure where distinct populations had on/off differences in selection regime, e.g. presence/absence of predatory fish in ponds and lakes, see also [9], [10], [43]. A number of empirical studies on different taxa have shown an inverse relationship between levels of gene flow, i.e. dispersal rate, among populations and the degree of adaptive divergence in defence traits, including both behavioural and morphological traits [44]–[46]. Dispersal potential among freshwater organisms varies considerably [47]. Snails depend on passive dispersal and thus should have limited dispersal ability compared to other freshwater invertebrates, e.g. insects with a winged adult stage. However, *R. balthica* is known to have a remarkably strong dispersal ability ([25]; cf. its former species name, *peregra*, and common name, wandering snail) and a study of snails incidence functions in the region (Brönmark unpublished mansuscript) suggest that it has a high dispersal potential (a supertramp [48]). A study of colonisation of benthic invertebrates into newly created wetlands [49] as well as molecular analyses [50] further confirms a high migration rate in this species. Thus, we suggest that the combination of a high dispersal rate [8], [11], [12] and the presence/absence of predation – fish or no-fish – in ponds and lakes in a metapopulation landscape that creates a fine grain environment has favoured the evolution of phenotypic plasticity in *R. balthica*.

## Supporting Information

Appendix S1 **Further details of the principal components analysis.**
(XLS)

## References

[1] Kats LB, Petranka JW, Sih A (1988). Antipredator defenses and the persistence of amphibian larvae with fishes.. Ecology.

[2] Lima SL, Dill LM (1990). Behavioral decisions made under the risk of predation: a review and prospectus.. Can J Zool.

[3] Crowl TA, Covich AP (1990). Predator-induced life-history shifts in a freshwater snail.. Science.

[4] Brönmark C, Miner JG (1992). Predator-induced phenotypical change in body morphology in crucian carp.. Science.

[5] Turner AM, Montgomery SL (2003). Spatial and temporal scales of predator avoidance: experiments with fish and snails.. Ecology.

[6] Tollrian R, Harvell DC (1999). The ecology and evolution of inducible defences.

[7] Levins R (1968). Evolution in Changing Environments.

[8] Hollander J (2008). Testing the grain-size model for the evolution of phenotypic plasticity.. Evolution.

[9] Lenormand T (2002). Gene flow and the limits to natural selection.. Trends Ecol Evol.

[10] Kawecki TJ, Ebert D (2004). Conceptual issues in local adaptation.. Ecol Lett.

[11] Via S, Lande R (1985). Genotype-environment interaction and the evolution of phenotypic plasticity.. Evolution.

[12] Sultan SE, Spencer HG (2002). Metapopulation structure favors plasticity over local adaptation.. Am Nat.

[13] Glöer P (2002). Die Süsswassergastropoden Nord- und Mitteleuropas.

[14] Brönmark C, Klosiewski SP, Stein RA (1992). Indirect effects of predation in a freshwater, benthic food chain.. Ecology.

[15] Brönmark C (1992). Leech predation on juvenile freshwater snails: effects of size, species and substrate.. Oecologia.

[16] Nyström P, Brönmark C, Granéli W (1999). Influence of an exotic and a native crayfish species on a littoral benthic community.. Oikos.

[17] Brönmark C, Malmqvist B (1986). Interactions between the leech *Glossiphonia complanata* and its gastropod prey.. Oecologia.

[18] Rundle SD, Brönmark C (2001). Inter- and intraspecific trait compensation of defence mechanisms in freshwater snails.. Proc R Soc Lon B.

[19] DeWitt TJ, Robinson BW, Wilson DS (2000). Functional diversity among predators of a freshwater snail imposes an adaptive trade-off for shell morphology.. Evol Ecol Res.

[20] Langerhans BR, DeWitt TJ (2002). Plasticity constrained: over-generalized induction cues cause maladaptive phenotypes.. Evol Ecol Res.

[21] Hoverman JT, Relyea RA (2007). How flexible is phenotypic plasticity? Developmental windows for trait induction and reversal.. Ecology.

[22] Hoverman JT, Relyea RA (2007). The rules of engagement: how to defend against combinations of predators.. Oecologia.

[23] Hoverman JT, Auld JR, Relyea RA (2005). Putting prey back together again: integrating predator-induced behavior, morphology, and life history.. Oecologia.

[24] DeWitt TJ, Sih A, Hucko JA (1999). Trait compensation and cospecialization in a freshwater snail: size, shape and antipredator behaviour.. Anim Behav.

[25] Hubendick B (1951). Recent *Lymnaeidae*, their variation, morphology, taxonomy, nomenclature and distribution.. Kongliga Svenska Vetenskaps-Akademiens Handlingar.

[26] Lam PKS, Calow P (1988). Differences in the shell shape of *Lymnaea peregra* (Müller) (Gastropoda: Pulmonata) from lotic and lentic habitats; environmental or genetic variance?. J Moll Stud.

[27] Wullschleger EB, Ward PI (1998). Shell form and habitat choice in *Lymnaea*.. J Moll Stud.

[28] Brönmark C (1985). Interactions between macrophytes, epiphytes and herbivores: an experimental approach.. Oikos.

[29] Brönmark C, Weisner SEB (1996). Decoupling of cascading trophic interactions in a freshwater, benthic food chain.. Oecologia.

[30] Hansson L-A, Brönmark C, Nilsson AP, Åbjörnsson K (2005). Conflicting demands on wetland ecosystem services: nutrient retention, biodiversity or both?. Freshw Biol.

[31] Iwata H, Ukai Y (2002). SHAPE: a computer program package for quantitative evaluation of biological shapes based on elliptic Fourier descriptors.. J Hered.

[32] R Development Core Team (2008). R: a language and environment for statistical computing.. http://cran.R-project.org.

[33] Wullschleger EB, Jokela J (2002). Morphological plasticity and divergence in life-history traits between two closely related freshwater snails, *Lymnaea ovata* and *Lymnaea peregra*.. J Moll Stud.

[34] Auld JR, Relyea RA (2010). Inbreeding depression in adaptive plasticity under predation risk in a fresh water snail.. Biol Let.

[35] Palmer AR (1979). Fish predation and the evolution of gastropod shell sculpture – experimental and geographic evidence.. Evolution.

[36] Relyea RA (2007). Getting out alive: how predators affect the decision to metamorphose.. Oecologia.

[37] Trussel GC (2000). Predator-induced plasticity and morphological trade-offs in latitudinally separated populations of *Littorina obtusata*.. Evol Ecol Res.

[38] Hollander J, Collyer ML, Adams DC, Johannesson K (2006). Phenotypic plasticity in two marine snails: constraints superseding life history.. J Evol Biol.

[39] Pigliucci M (2001). Phenotypic Plasticity: Beyond Nature and Nurture.

[40] Price TD, Qvarnström A, Irwin DE (2003). The role of phenotypic plasticity in driving genetic evolution.. Proc R Soc Lon B.

[41] Hollander J, Butlin RK (2010). The adaptive value of phenotypic plasticity in two ecotypes of a marine gastropod.. BMC Evol Biol.

[42] van Tienderen PH (1991). Evolution of generalists and specialists in spatially heterogeneous environments.. Evolution.

[43] Crispo E (2008). Modifying effect of phenotypic plasticity on interactions among natural selection, adaptation and gene flow.. J Evol Biol.

[44] Hendry AP, Taylor EB (2004). How much of the variation in adaptive divergence can be explained by gene flow? – An evaluation using lake-stream stickleback pairs.. Evolution.

[45] Nosil P, Crespi BJ (2004). Does gene flow constrain adaptive divergence or vice versa? A test using ecomorphology and sexual isolation in *Timema cristinae* walking-sticks.. Evolution.

[46] Lind MI, Johansson F (2007). The degree of adaptive phenotypic plasticity is correlated with the spatial environmental heterogeneity experienced by island population of *Rana temporaria*.. J Evol Biol.

[47] Bilton DT, Freeland JR, Okamura B (2001). Dispersal in freshwater invertebrates.. Ann Rev Ecol Syst.

[48] Diamond JM, Cody ML, Diamond JM (1975). Assembly of species communities.. Ecology and Evolution of Communities.

[49] Ekologgruppen (2002). Biologisk mångfald i dammar.. Bottenfauna (in Swedish).

[50] Evanno G, Castello E, Goudet J (2006). Evolutionary aspects of population structure for molecular and quantitative traits in a fresh water snail *Radix balthica*.. J Evol Biol.

